# Reducing Liver Fat by Low Carbohydrate Caloric Restriction Targets Hepatic Glucose Production in Non-Diabetic Obese Adults with Non-Alcoholic Fatty Liver Disease

**DOI:** 10.3390/jcm3031050

**Published:** 2014-09-22

**Authors:** Haoyong Yu, Weiping Jia, ZengKui Guo

**Affiliations:** 1 Department of Endocrinology and Metabolism, Shanghai Jiaotong University Affiliated Sixth People’s Hospital, Shanghai Diabetes Institute, Shanghai Clinical Centre of Diabetes, Shanghai 200233, China; E-Mails: yuh@206.com (H.Y.); wpjia@sjtu.edu.cn (W.J.); 2 Endocrine Research Unit, Division of Endocrinology, Diabetes, Metabolism and Nutrition, Department of Internal Medicine, Mayo Foundation, 5-194 Joseph, Rochester, MN 55905, USA

**Keywords:** obesity, caloric restriction, liver fat, hepatic glucose production, glucose disposal

## Abstract

Non-alcoholic fatty liver disease (NAFLD) impairs liver functions, the organ responsible for the regulation of endogenous glucose production and thus plays a key role in glycemic homeostasis. Therefore, interventions designed to normalize liver fat content are needed to improve glucose metabolism in patients affected by NAFLD such as obesity. Objective: this investigation is designed to determine the effects of caloric restriction on hepatic and peripheral glucose metabolism in obese humans with NAFLD. Methods: eight non-diabetic obese adults were restricted for daily energy intake (800 kcal) and low carbohydrate (<10%) for 8 weeks. Body compositions, liver fat and hepatic glucose production (HGP) and peripheral glucose disposal before and after the intervention were determined. Results: the caloric restriction reduced liver fat content by 2/3 (*p* = 0.004). Abdominal subcutaneous and visceral fat, body weight, BMI, waist circumference and fasting plasma triglyceride and free fatty acid concentrations all significantly decreased (*p* < 0.05). The suppression of post-load HGP was improved by 22% (*p* = 0.002) whereas glucose disposal was not affected (*p* = 0.3). Fasting glucose remained unchanged and the changes in the 2-hour plasma glucose and insulin concentration were modest and statistically insignificant (*p* > 0.05). Liver fat is the only independent variable highly correlated to HGP after the removal of confounders. Conclusion: NAFLD impairs HGP but not peripheral glucose disposal; low carbohydrate caloric restriction effectively lowers liver fat which appears to directly correct the HGP impairment.

## 1. Introduction

The links between obesity and insulin resistance includes the roles of lipid deposition in non-adipose tissues, such as skeletal muscle, islets and liver [1,2], collectively called ectopic fat. The adverse effects of ectopic fat on tissue functions are thought to be mediated by lipotoxicity among other mechanisms [3]. Peripherally, lipotoxicity is manifested as attenuated skeletal muscle insulin sensitivity, thereby impairing its ability to take up glucose. By comparison, in the liver, lipotoxicity impairs hepatic glucose metabolism by reducing its suppression of hepatic glucose production (HGP) (as HGP accounts for the bulk of endogenous glucose production, the term HGP is interchangeably used with EGP throughout this report). Non-alcoholic fatty liver disease (NAFLD) comprises a spectrum of conditions extending from simple liver steatosis (fatty infiltration of liver parenchymal tissue) to more severe liver disease such as steatohepatitis (ectopic fat associated with inflammations) [4,5]. NAFLD impairs suppression of glucose production and causes hyperglycemia [6]. NAFLD is highly prevalent in individuals with type 2 diabetes or/and obesity and contributes to hepatic insulin resistance. Thus, hepatic ectopic fat is likely a critical factor modulating HGP. This is probably one reason why NAFLD has a high prevalence in type 2 diabetes and obesity.

Caloric restriction (CR) is one of a few available effective remedies for improving insulin sensitivity and glucose metabolism. Studies have demonstrated potentially beneficial effects of CR on liver fat accumulation and systemic lipid profile [7]. However, studies on the effects of CR on liver fat and glucose metabolism in humans are limited. Especially, data are lacking relevant to the relationship between liver fat and hepatic glucose production and peripheral glucose disposal. In this report, we present the results from clinical studies in obese adults with increased liver fat content who are treated by aggressive caloric restriction in an attempt to examine the effects on hepatic triglyceride accumulation, hepatic glucose production and peripheral glucose disposal.

## 2. Methods and Materials

### 2.1. Study Participants

Eight participants (3 men, 5 women, 21–52 years old, BMI 32 kg/m^2^) participated the study at the Department of Endocrinology and Metabolism, Shanghai Jiaotong University Affiliated Sixth People’s Hospital. The participants underwent a general health examination and body and regional fat and liver fat content measurements (below). Individuals who are obese (BMI >30 kg/m^2^), non-diabetic with a liver fat content of >5.6%, with or without impaired fasting glucose or impaired glucose tolerance were selected to participate in this study. Exclusion criteria include women in pregnancy or to-be-pregnant, in lactation or post-menopausal, in use of any prescription medications within the previous 2 months, on weight loss programs by dieting or pills during the past 6 months, consuming alcohol of >20 g a day, tobacco use within 6 month, with cardiovascular or endocrine disease history, hypertension history or current elevated blood pressure (systolic blood ≥150 mm Hg, diastolic blood ≥90 mm Hg), with diabetes, acute or chronic infections, acute or chronic liver, kidney or gastrointestinal or other organ diseases. The study was approved by the institutional review board of Shanghai Jiao Tong University Affiliated Sixth People’s Hospital in accordance with the principles of the Helsinki Declaration II. Written informed consent was obtained from each participant.

### 2.2. Study Protocol

One week before initiation of the study, the participants were asked to maintain their dietary habits and energy intake. At baseline and again after the 8-week experiment, a modified (see below) 75-g oral glucose tolerance test was administered in each participant. At week 0, 4 and 8, anthropometric parameters, body composition and metabolic indices were measured as described below.

### 2.3. The Treatment-Dietary Intervention

All participants were given individualized instructions how to take a very low carbohydrate diet where energy intake was restricted to less than 800 kcal/day (carbohydrate intake <20 g/day). The daily meals were as follows: a cup of soybean milk (200 mL) and a boiled egg at breakfast; a nutrition bar (106 kcal: 2.8 g carbohydrate, 11.2 g protein, 5.6 g fat; Nutriease Health Technology Co., Ltd., Hangzhou, China), non-starch vegetables (<200 kcal), and 50 g protein (beef, lean pork, skinned chicken, fish) for lunch and dinner. Supplementation of multivitamins and minerals was provided daily. The participants were encouraged to drink at least 1.8 L of water daily, and asked to maintain their habitual physical activity levels. Regular telephone contact by nutritionists was provided for support. After the 8-week intervention, a 1-week recovery period on isocaloric intake was allowed to recover from the low energy intake state before the participants resume their usual eating habits.

### 2.4. Body Composition Measurements

Whole body skeletal muscle mass and fat mass were measured by bioimpedance using a multifrequency impedance plethysmography analyzer (InBody 720, Biospace, South Korea). Participants stood on the electrodes embedded in a scale platform of the analyzer after the feet bottom was wiped with a proprietary electrolyte sheet (for electric conduction). Then, they were asked to stand upright while grasping the handles of the analyzer to make contact with four pairs of electrodes (octapolar format). Electric resistance was measured at five frequencies (1, 50, 250, 500 and 1 kHz) and reactance at three frequencies (5, 50 and 250 kHz). Total body water (TBW) was estimated from area, volume, length, impedance and a constant of specific resistivity. Fat-free mass was estimated by dividing TBW by 0.73. Fat mass and skeletal muscle mass as a percentage of body weight were calculated by a built-in computer software [8].

### 2.5. Measurement of Regional Fat

Magnetic Resonance Imaging (MRI) was used to determine visceral fat and abdominal subcutaneous fat (Philips Achieva 3.0T MRI system, Philips Medical Systems, Eindhoven, Netherlands) using standard array coils with the participants in a supine position. Breath-hold images were centered on the L4–L5 intervertebral disc using standard localizer images with the following parameters: TR 4 ms, TE 2 ms, number of slices 12, slice thickness 8 mm, image matrix 256 × 256, and field-of-view 500 × 500 mm. The four slices that were best aligned with the L4–L5 disc were analyzed by Slice Omatic 5.0 software (Escape Medical Viewer V3.2) to define visceral and abdominal subcutaneous fat [9] by fitting a spline curve to points on the border of the abdominal subcutaneous and visceral regions. Non-fat regions within the visceral region were also outlined with a spline fit and subtracted from the total visceral region [10].

### 2.6. Determination of Liver Fat Content

Liver MRI and *in vivo* single-voxel proton magnetic resonance spectroscopy (^1^H MRS) were performed using a Philips Achieva 3.0T system (Philips Medical Systems, Eindhoven, Netherlands) equipped with an 8-channel phase coil. Anatomical T1-weighted spin-echo MR images were obtained using the following parameters: repetition time (TR) 550 ms; echo time (TE) 10 ms; flip angle 60; field of view (FOV) 21 cm; slice thickness 3 mm; slice spacing 0.1 mm. ^1^H-MRS was used to measure hepatic metabolites. 2D-Spin-echo images in the coronal and sagittal regions were obtained for image-guided localization of voxel of interest (VOI) for spectroscopic data acquisition. Then, single-voxel MRS was performed by a stimulated echo acquisition mode sequence using the following parameters: TE 20 ms; TR 1500 ms; VOX 15 × 15 × 15 mm; total number of points 128; total number of average 64. Finally, eight-step phase cycling was used to suppress unwanted signals or artifacts. Signal intensities of the water peak at 4.7 ppm (*S*w) and the intrahepatocellular fat peak at 1.2 ppm (*S*f) were quantified. Liver fat % (LFP) is then calculated: *S*f/(*S*f + *S*w) × 100% [11]. A liver fat of 5.56% is used as cutoff value for diagnosing non-alcoholic fatty liver disease [12].

### 2.7. Determination of Hepatic Glucose Production and Peripheral Glucose Disposal

Before and after the 8-week dietary intervention, each participant underwent an isotope-assisted oral glucose tolerance test as described previously (iOGTT) [13]. Briefly, 2 g of 6,6-d_2_-glucose were included in the oral glucose (Isotec, Miamisburg, OH, USA) with the total dose remained at 75 g. Blood samples were collected from an antecubital vein at 0 (before the glucose drink) and at 30, 60, 90, 120, 150 and 180 minutes after the drink. Then, the participants were dismissed from the hospital. The blood was processed to separate plasma for glucose, insulin and blood chemistry measurements. In addition, the enrichment of d_2_-glucose was determined by GC-MS (HP model 6890/5972). The results allowed the quantitation of the portion of plasma glucose that originates from the orally administered glucose. The peripheral glucose disposal is quantified by the reciprocal of the orally-derived glucose based on the inverse relationship between the two parameters (*i.e.*, circulating orally-derived glucose is a function of glucose uptake primarily by skeletal muscle). The difference between the orally-derived plasma glucose and the total plasma glucose is HGP [13]. The area under the curve (AUC) for HGP and glucose uptake are calculated as the quantities of the two parameters for data analysis.

### 2.8. Statistics

Data are presented in mean ± SE unless as indicated otherwise. Comparisons before and after caloric restriction is performed using paired Student’s *t*-test at one tail. An alpha value of 0.05 is used as the criteria for statistical significance. The correlation between improvement of hepatic glucose production or glucose disposal and the reduction of hepatic fat content is performed using linear regression analysis. Multivariate regression was used for covariance analysis.

## 3. Results

Table 1 shows the effects of very low carbohydrate caloric restriction on the physical characteristics and metabolic parameters in human obesity. At the end of the 8-week intervention, body weight was reduced by 6.8 kg on average, or 7% of the pre-treatment weights (*p* = 0.001).

**Table 1:** The effects of very low carbohydrate-based caloric restriction in obese adults.

Parameters	Before	After	*p*
body weight, kg	87.7 ± 3.8	80.9 ± 3.0	0.001
BMI, kg/m^2^	32.0 ± 0.7	29.5 ± 0.4	0.0004
waist, cm	100 ± 2.9	95.0 ± 1.2	0.04
TG, mmol/L	1.85 ± 0.59	1.11 ± 0.34	0.04
FFA, mmol/L	0.73 ± 0.03	0.57 ± 0.04	0.01
HDL, mmol/L	1.28 ± 0.14	1.24 ± 0.11	0.29
LDL, mmol/L	2.98 ± 0.29	3.14 ± 0.27	0.31
ASQ fat, cm^2^	321 ± 31	244 ± 21	0.005
visceral fat, cm^2^	99 ± 10	65 ± 4	0.007
liver fat, %	28.8 ± 7.2	9.5 ± 2.7	0.004
FPG, mmol/L	5.41 ± 0.23	5.41 ± 0.15	0.4
2hPG, mmol/L	8.0 ± 0.8	7.4 ± 0.4	0.1
FINS, pmol/L	127 ± 38	85 ± 11	0.1
2hINS, pmol/L	762 ± 179	605 ± 100	0.2
HOMA-IR	4.7 ± 1.6	2.9 ± 0.4	0.14

Values are mean ± SE; *n* = 8 (3/5, m/f), age 37 ± 3; ASQ, abdominal subcutaneous; FPG, fasting plasma glucose; 2hPG, 2-hour-plasma glucose; FINS, fasting plasma insulin; 2hINS, 2-hour-plasma insulin.

The body weight changes were mainly a result of shrinking in body fat, especially the visceral fat which decreased by 1/3 (*p* = 0.007). The abdominal subcutaneous fat was also decreased (24%, *p* = 0.005). These changes were accompanied by a shortening of the waist line by 5 cm (*p* = 0.04). Due to these changes in body composition, the classification of the group as a whole improved from obesity to overweight based on the universal cutoff value of BMI at 30 kg/m^2^ (*p* = 0.0004). 

Plasma TG (*p* = 0.04) and FFA (*p* = 0.01) concentrations decreased significantly whereas HDL and LDL did not (*p* > 0.05). Fasting plasma glucose remained the same as before the treatment. The 2-hour plasma glucose decreased modestly without reaching statistical significance (*p* = 0.1). Fasting and 2-hour insulin concentrations were lowered by 20%–34%, suggesting improved insulin sensitivity.

### 3.1. Liver Fat and Organ-Specific Glucose Metabolism

The most remarkable change after the very low carbohydrate caloric restriction was a reduction of liver fat content by 2/3 (*p* = 0.004) (Table 2). The liver fat decreased continuously as measured at the 4th (data not shown) and 8th week of treatment but only the 8th week values were statistically significant. At the end of the period (8th week), the decrease in liver fat was associated with a significant decrease in HGP during the oral glucose tolerance test (*p* = 0.002), suggesting improved sensitivity of HGP to insulin suppression. In contrast, peripheral glucose uptake was not affected (*p* = 0.3, Table 2), indicating that the peripheral insulin sensitivity was not improved. Thus, the improvement in insulin sensitivity is largely limited to the liver.

**Table 2:** Effects of caloric restriction on liver fat and HGP and glucose disposal in obese adults.

Statistics	Liver Fat %			HGP			Glucose Disposal		
	before	after	delta *^p^*	before	After	delta *^p^*	before	after	delta *^p^*
mean ± SE	28.8 ± 7.2	9.5 ± 2.7	67% ^0.004^	762 ± 82	598 ± 54	22% ^0.002^	748 ± 144	822 ± 121	10% ^0.3^
*r* (liver fat %)	1	1	1	0.84	0.74	0.77	0.15	0.05	0.14
*p*				0.01	0.03	0.02	0.7	0.9	0.74
-BW				0.02	0.03	0.02	0.7	0.8	0.7
-VF				0.03	0.02	0.01	0.6	0.7	0.7

HGP, hepatic glucose production; HGP and glucose disposal are in AUC measured using iOGTT as described in Methods; Delta, % before and after intervention differences with the *p* values shown as superscript; *r*, correlation coefficients with liver fat (the *p* values are shown in the row directly below; -BW, -VF: the same *p* values after removal of body weight, visceral fat, respectively, by multiple regression analysis).

### 3.2. The Relationship between Liver Fat Content and Hepatic Glucose Production

**Figure 1:**
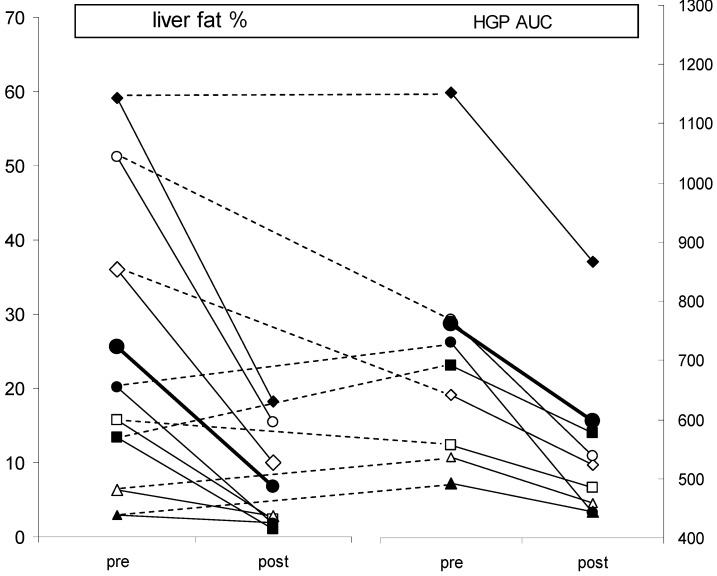
The correlation between the changes in liver fat % (left panel) and the corresponding changes in HGP (right panel) as a result of caloric restriction in obese adults; the solid lines connect pre- and post-treatment values; the broken lines connect the same subjects, with same symbols, to show that the ranking of the two changes are the same in six of the eight subjects, suggesting a possibility of cause-effect relationship between the two parameters.

Figure 1 shows that in six of the eight subjects, the ranking of magnitude of the reductions in liver fat and HGP is the same. In other words, the reduction in HGP is a function of the reduction in liver fat.

**Figure 2:**
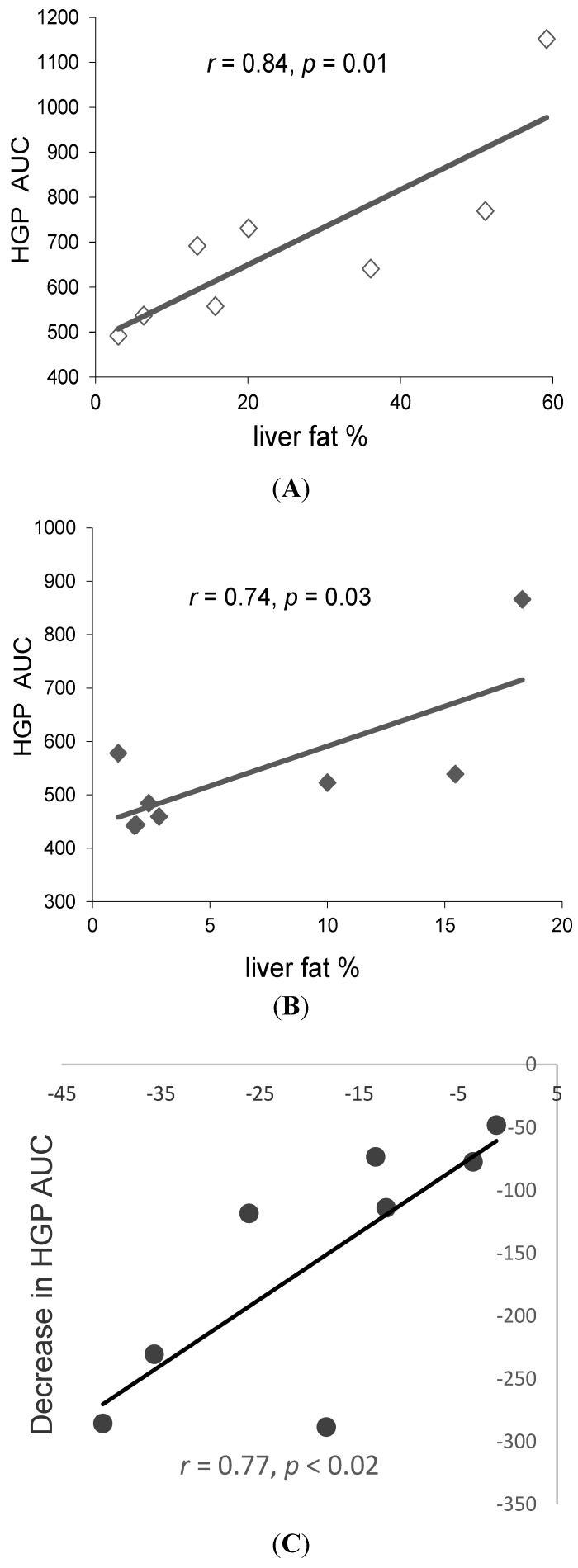
Correlations between liver fat % and post-load HGP in obese adults before (**A**) and after (**B**) the 8 week caloric restriction, and the correlation between their changes at the end of the intervention (**C**); liver fat is measured by MRS. HGP is quantified by iOGTT (see Methods for detail).

Furthermore, the liver fat content pre- and post-treatment and its reductions (delta) after the treatment are all highly correlated with HGP (correlation coefficients 0.74–0.84, Figure 2).

The results from multiple linear regression analysis showed that liver fat content is the only independent variable significantly correlated to HGP after correction for body weight, BMI, plasma TG or visceral fat (Table 2). In contrast, the correlations of peripheral glucose uptake with liver fat were weak (*r* = 0.05–0.15) and not statistically significant (Table 2).

The improved suppression of HGP was not due to increased insulin actions because both the basal and 2-hour plasma insulin were lower compared to the pre-treatment values. The whole body insulin resistance is not significantly improved as assessed by HOMA-IR (Table 1). Therefore, the improved suppression of HGP appeared to be mainly a result of local, *i.e.*, intrahepatic, improvement in insulin action attributable to the lowered liver fat. Corrected for the change in visceral fat and body weight, the change in liver fat remained as a strong predictor of decreases in HGP (Table 2).

### 3.3. Interindividual Variability

This is the first study whereby using the iOGTT technique, hepatic glucose production can be determined and evaluated in relation to other physical or metabolic variables in a clinical setting. This allowed one to classify the systemic glucose intolerance in a patient as liver type (hepatic insulin resistance), muscle type (peripheral insulin resistance) or mixed type, *i.e.*, both the liver and muscle are insulin resistant and responsible for the glucose intolerance [13]. Before the intervention, four of the eight participants were classified as liver type (impaired HGP) (Table 3). Their liver fat content was 36% on average. The intervention reduced it to 9.2%, a level slightly higher than the normal value of 5.6% [12]. This large drop in liver fat is accompanied by a 27% decrease in HGP which led to the reclassification for three of these four participants (*i.e.*, no longer hepatic insulin resistant), except ZT who remained liver type. Among the three reclassified, only one was both IFG (impaired fasting glucose) and IGT (impaired glucose tolerance) before the intervention (WQ). After treatment, IFG regressed to NFG (normal fasting glucose) but IGT remained. This participant was also the only one among the eight who was classified muscle type (*i.e.*, a mixed type participant). The intervention increased glucose uptake slightly so that muscle type is reverted to normal.

**Table 3:** Inter-patient variability in the effects of low carbohydrate caloric restriction in obesity.

Patient ID	IFG		IGT		Plasma insulin		Impaired HGP		Impaired Rd	
	pre	post	pre	post	basal	post-load	pre	post	pre	post
ZT	yes	yes	yes	yes	↓	↓↓^†^	yes	yes	no	no
GL	no	no	no	no	↑	↑-	no	no	no	no
WZ	no	no	no	no	↑	↓-	no	no	no	no
HT	no	no	no	no	↑	↑-	yes	no	no	no
FJ	no	no	no	no	↓	↓-	yes	no	no	no
JLL	no	no	no	no	−	↓-	no	no	no	no
WQ	yes	no	yes	yes	↓	↓↓^†^	yes	no	yes	no
JLY	no	no	no	no	−	↓↓^†^	no	no	no	no

IFG, impaired fasting glucose; IGT, impaired glucose tolerance; post-load insulin is the sum of plasma insulin for all the sampling time points (arrows: increase or decrease compared to pre-treatment: -, *p* > 0.1; ^†^, *p* < 0.01); −, No change.

Even though not reclassified, like the others, participant ZT had large decrease in liver fat (59% to 18%) coincided with a 25% decrease in HGP. However, the HGP remained too high to be reclassified. This was also the only participant who had IFG and IGT both before and after the intervention. However, glucose disposal was normal before and after. Thus, liver but not the periphery was responsible for the persistent glucose intolerance in this participant. The post-load plasma insulin reduced to 53% of the pre-treatment level. So, it is unclear whether the high HGP was also due to a lack of post-prandial insulin response or only due to hepatic insulin resistance.

The other four participants had minor impairment in HGP and thus were not liver type, nor muscle type. None of them were IFG or IGT, before or after. Their liver fat decreased from 15.3% to 4.3% with a 14% reduction in HGP. Based on these observations, it appears that in non-diabetic, obese participants with NAFLD, the liver, rather than the periphery, is more likely the site of insulin resistance. This is expected considering the intrahepatic lipotoxicity from the ectopic fat. On the other hand, the data showed that there are considerable inter-individual variabilities that make it impractical to conclude so in a general sense. In fact, this is what “individualized glycemic control” means, that is, each patient is examined and treated separately. This is made possible by using iOGTT by differentiating the liver *versus* the periphery as the locale of insulin resistance and, therefore, the target for treatment. 

## 4. Discussion

Nonalcoholic fatty liver disease is the most prevalent form of liver diseases in the United States. It occurs in 20%–30% of Americans, approaching the prevalence rate of obesity, consistent with the fact that the occurrence of NAFLD in obesity is high. NAFLD is closely linked to hepatic insulin resistance. In some individuals it can lead to steatohepatitis, cirrhosis, and even cancer [14]. Increased hepatic glucose production, or impaired insulin suppression of it, is one of the two pathways (endogenous glucose production and glucose disposal) that are responsible for hyperglycemia, and thus NAFLD promotes type 2 diabetes.

The finding from this study showed that a significant benefit of low carbohydrate caloric restriction is reduced adiposity, including reduced body fat due to shrunk abdominal subcutaneous and visceral fat. These changes, not muscle, underlie the decreases in body weight and BMI. The most remarkable change is the 2/3 reduction in liver fat content accompanied by a 40% decrease in fasting plasma TG concentration, likely a result of reduced VLDL secretion. This reduction of liver fat is likely related to the nutrient composition of the diet used for the intervention, *i.e.*, low carbohydrate (<20 g CHO, daily energy intake 800 kcal). Although not directly tested in the present study, the previous studies indicated that low carbohydrate CR is more effective than low fat CR for weight loss, especially for reducing liver fat [15]. In healthy individuals, the contribution of *de novo* lipogenesis (DNL) to tissue triglycerides is <5% [16,17], even in obese humans [18]. NAFLD [19] may increase this contribution but it remains quantitatively minor. Combined with the low CHO intake in this study, DNL played little role in the changes of liver fat in these participants. Plasma FFA became the main source of precursor for TG synthesis [16,17,20]. However, under CR the system is in a catabolic state where fatty acids are oxidized for ATP production, not for esterification. Meanwhile, the shrinking of adipose tissues reduced the amounts of fatty acids exported via lipolysis. These are consistent with the decrease in plasma FFA and may have caused the liver fat reduction. Because fatty acid carbons biochemically do not contribute to glucose synthesis, the availability of both glucose and fatty acids is limited as the substrates for glycerol and acyl moieties of TG molecules, hence reduced TG synthesis. Gluconeogenesis or/and glyceroneogenesis [21] therefore probably provided much of the needed glycerol. However, the participants did not lose the mass of skeletal muscle, suggesting lean tissue degradation was limited as a way to provide the needed gluconeogenic substrates. This left glycerol as the main substrate for gluconeogenesis. However, reduced lipolysis also means reduced release of glycerol from adipose tissues, especially considering that skeletal muscle is also a sink of plasma glycerol [21]. Thus, overall the substrates for both moieties of TG molecules were limited with a low carbohydrate diet. In contrast, with a low fat diet, fatty acids can be easily generated from glucose carbons via DNL. As such, TG moieties are also not limiting and thus TG accumulation can continue. Understanding whether and how low CHO caloric restriction is more effective than low fat diets will help in selecting effective strategies for reducing liver fat, and requires further investigations.

The main beneficiary of lowering liver fat appears to be the enhanced postprandial suppression of hepatic glucose production. The post-load HGP in this study is highly correlated with liver fat content both before and after the intervention (Table 2). After the intervention, corresponding to the reduction in liver fat is a significant improvement in the suppression of HGP. Multiple linear regression analysis indicated that liver fat content is the only independent variable significantly correlated to HGP after the removal of the confounders including body weight, BMI, fasting TG and visceral fat. Such strong correlations between liver fat and HGP suggest a possibility that liver fat is an entity that is causally related to post-prandial HGP. If this is true, increased liver fat accumulation would raise HGP; conversely, a decreased liver fat accumulation would do just the opposite. This is exactly what is observed in the present study. This hypothesis is consistent with the lack of extrahepatic factors involved in improving HGP. In addition to those factors mentioned above (body weight, BMI, visceral fat and plasma TG), other extrahepatic factors include plasma insulin and glucose. However, these variables could not have contributed to the improvement in HGP as insulin decreased while glucose hardly changed. Accordingly, it is proposed that liver fat directly modulates postprandial HGP in human obesity. As a state of energy excess in the form of high adiposity, this issue is especially relevant to obesity and diabesity [22]. A role for tissue fat in regulating hepatic glucose metabolism is also evident in severe obesity in which gastric bypass surgery greatly lowered HGP [23]. Thus, HGP-suppressing effect of low carbohydrate caloric restriction may specially benefit patients with insulin resistance localized in the liver [13]. The observed lack of effect of liver fat on peripheral glucose disposal needs confirmation by additional studies. It appears, however, that the periphery is insensitive to the secondary effect, if any, of the reduced body fat and regional fat so that the post-intervention glucose disposal remained uncorrelated to liver fat. In contrast, the significant effects of liver fat on hepatic glucose production cannot be ruled out for a possibility of the secondary effects. Clarification of this issue is extremely interesting and important. If it can be proven that such secondary effects are absent, strategies to reduce liver fat alone without effects on extrahepatic factors would be able to improve HGP in obesity and therefore slow down or prevent from progressing to type 2 diabetes.

A limitation of the present study is the small sample size. This is mainly for the consideration that it is a proof-of-principle study designed to evaluate the feasibility of identifying the organ(s) with impaired glucose metabolism, and the severity thereof, in each patient using the clinically feasible isotope-assisted oral glucose tolerance test technique (iOGTT). The results confirmed our hypothesis that the hepatic glucose metabolism is exquisitely correlated to liver fat content. The encouraging results obtained from the study support further investigations in larger scale using the approach to gain new insights into the relationship between liver fat and organ-specific glucose metabolism. The use of a small sample size may also “miss” some of the effects of the intervention by failing to detect them at statistical significance. This may be compounded by potential gender difference in metabolic response to the treatment. Without measuring the rates of fatty acid and glycerol flux (lipolysis), the discussions above are speculations only. Thus, the exact mechanisms underlying the remarkable reduction of liver fat are unknown. At the regulatory levels, the potential mechanisms may include altered signaling and transcriptions. Accelerated HGP in the face of liver fat accumulation includes increased gluconeogenesis [24]. Conversely, the regression of liver fat content suggests a removal of the lipo-stimuli or lipotoxicity, limiting gluconeogenesis and hence reduced HGP. Diacylglycerol acyltransferase (DGAT), the rate-limiting enzyme for the final step of TG synthesis, is likely involved [22]. In animals, caloric restriction was shown to involve enhanced SIRT1 signaling in the liver [25]. A down regulation of SREBP, the master transcriptional regulator for enzymes involved in lipid anabolism, may also be involved in caloric restriction-induced liver fat reduction. By comparison, carbohydrate response element-binding protein (ChREBP) or acetyl-CoA carboxylase may be less relevant given the low carbohydrate intake. On balance, the present study is strengthened by the use of the state-of-the-art MRS technology for liver fat measurement. The highly elevated liver fat contents in these obese individuals (BMI > 30 mg/m^2^) are consistent with that of an earlier study on similar ethnic groups (Northeast Asians) where BMI is identified as the strongest predictor of liver fat content [26]. Furthermore, the present study is the first to clinically identify the organs (liver *versus* muscle) that is responsible for impaired glucose tolerance in individual patients.

## 5. Conclusions

The finding from this investigation confirmed that low carbohydrate-based caloric restriction, even for a relatively short duration (~2 months), is effective in reducing liver fat in obesity with non-alcoholic fatty liver disease. The novelty of the finding is that the reduction in liver fat content appears to directly decrease postprandial hepatic glucose production in this population. By using the iOGTT technique, postprandial hepatic glucose production and peripheral glucose disposal can be assessed in individual patients, clinically. This enables the differentiation of liver *versus* periphery as being the locale responsible for glucose intolerance. By comparing the pre- and post-intervention differences, it becomes possible to determine whether a given intervention strategy improves hepatic or skeletal muscle glucose metabolism. The accuracy of this approach requires further investigations.
